# RGS20 promotes non-small cell lung carcinoma proliferation via autophagy activation and inhibition of the PKA-Hippo signaling pathway

**DOI:** 10.1186/s12935-024-03282-9

**Published:** 2024-03-02

**Authors:** Xiaoyan Ding, Xiaoxia Li, Yanxia Jiang, Yujun Li, Hong Li, Lipeng Shang, Guilin Feng, Huhu Zhang, Ziyuan Xu, Lina Yang, Bing Li, Robert Chunhua Zhao

**Affiliations:** 1 School of Basic Medicine, Institute of Stem Cell and Regenerative Medicine, Qingdao University, Qingdao, China; 2 Department of Pathology, The Affiliated Hospital of Qingdao University, Qingdao, China; 3 Institute of Basic Medical Sciences, Chinese Academy of Medical Sciences, School of Basic Medicine, Peking Union Medical College, Beijing, 100005 China; 4 School of Life Sciences, Shanghai University, Shanghai, China

**Keywords:** NSCLC, RGS20, Proliferation, Autophagy, PKA-Hippo signaling pathway

## Abstract

**Background:**

Novel therapeutic targets are urgently needed for treating drug-resistant non-small cell lung cancer (NSCLC) and overcoming drug resistance to molecular-targeted therapies. Regulator of G protein signaling 20 (RGS20) is identified as an upregulated factor in many cancers, yet its specific role and the mechanism through which RGS20 functions in NSCLC remain unclear. Our study aimed to identify the role of RGS20 in NSCLC prognosis and delineate associated cellular and molecular pathways.

**Methods:**

Immunohistochemistry and lung cancer tissue microarray were used to verify the expression of RGS20 between NSCLC patients. CCK8 and cell cloning were conducted to determine the proliferation ability of H1299 and Anip973 cells in vitro. Furthermore, Transcriptome sequencing was performed to show enrichment genes and pathways. Immunofluorescence was used to detect the translocation changes of YAP to nucleus. Western blotting demonstrated different expressions of autophagy and the Hippo-PKA signal pathway. In vitro and in vivo experiments verified whether overexpression of RGS20 affect the proliferation and autophagy of NSCLC through regulating the Hippo pathway.

**Results:**

The higher RGS20 expression was found to be significantly correlated with a poorer five-year survival rate. Further, RGS20 accelerated cell proliferation by increasing autophagy. Transcriptomic sequencing suggested the involvement of the Hippo signaling pathway in the action of RGS20 in NSCLC. RGS20 activation reduced YAP phosphorylation and facilitated its nuclear translocation. Remarkably, inhibiting Hippo signaling with GA-017 promoted cell proliferation and activated autophagy in RGS20 knock-down cells. However, forskolin, a GPCR activator, increased YAP phosphorylation and reversed the promoting effect of RGS20 in RGS20-overexpressing cells. Lastly, in vivo experiments further confirmed role of RGS20 in aggravating tumorigenicity, as its overexpression increased NSCLC cell proliferation.

**Conclusion:**

Our findings indicate that RGS20 drives NSCLC cell proliferation by triggering autophagy via the inhibition of PKA-Hippo signaling. These insights support the role of RGS20 as a promising novel molecular marker and a target for future targeted therapies in lung cancer treatment.

**Supplementary Information:**

The online version contains supplementary material available at 10.1186/s12935-024-03282-9.

## Introduction

Non-small cell lung cancer (NSCLC) is the leading cause of cancer-related deaths worldwide [1] and include adenocarcinoma, squamous cell carcinoma, and large cell carcinoma [2]. With the advancement of molecular-targeted therapies, patients with NSCLC have seen significant treatment improvements over the past decade [3–5]. Further, inhibitors of kinases, such as epidermal growth factor receptor (EGFR) signaling [6], are in routine clinical use. However, resistance to current treatments often emerges within 2–3 months from the start of therapy in the majority of patients with advanced NSCLC [7]. Therefore, the discovery of novel biomarkers associated with NSCLC development has emerged as a prominent area of research, especially for developing more targeted and personalized treatment approaches.

Regulators of G protein signaling (RGS) proteins exert significant influence on the functions of G protein-coupled receptors (GPCRs) [8]. Among mammalian RGS proteins, RGS17 binds to and activates the GTPase activity of G (i/o), G (z), and G (q), thereby inhibiting GPCR signaling [9]. Another member, RGS20, shares about 62% similarity with RGS17 [10] and has been implicated in the development of various malignant tumors. For example, it is linked to the proliferation and migration of penile cancer [9], bladder cancer [11] and oral squamous cell carcinoma [12]. Further, high RGS20 expression correlates significantly with the progression and prognosis of triple-negative breast cancer and renal cancer [13, 14]. Considering the involvement of RGS20 in various cancers, it is surprising that the role of RGS20 in NSCLC remains largely unexplored. A recent study reported that RGS20 promotes cell aggregation and migration [10]. However, functional and prognostic analyses, as well as the molecular mechanisms of RGS20 in NSCLC still remain poorly understood.

In this study, we first examined the expression of RGS20 in clinical tumor samples of lung adenocarcinoma and squamous cell carcinoma, and explored the relationship between its expression level and prognosis. Next, we investigated the effects of overexpression or knockdown of RGS20 on cell proliferation and autophagy in NSCLC cells. Furthermore, transcriptomic sequencing was performed to find the key pathways involved in the role of RGS20. At last, the xenograft nude mouse model was used to confirm the role of RGS20 in NSCLC in vivo.

## Materials and methods

### Patient specimens and tissue microarrays

A total of 126 pairs of paraffin sections of lung adenocarcinoma and corresponding normal lung tissues were obtained from the Affiliated Hospital of Qingdao University, China. All samples were sequenced by NGS to exclude *egfr*, *alk*, *ros1*, *braf*, *her-2*, *kras*, *nras*, *pik3ca*, *ret* or *met* gene mutations. A human lung squamous carcinoma tissue microarray consisting of 75 pairs of primary lesions with matched normal adjacent control tissues (HLug-Squ150Sur-02) was purchased from Xinchao Biotechnology Co., Ltd. (Shanghai, China). Patient consent and ethical approval were obtained from the Institutional Research Ethics Committee (Ethical approval number: QYFY WZLL 27,991, SHYJS-CP-1,904,009).

### Cell lines and reagents

NSCLC cell lines (H1299, Anip973) were maintained in RPMI Medium 1640 (Gibco, Waltham, MA, USA) supplemented with 1% penicillin-streptomycin (NCM Biotech, Suzhou, China) and 10% heat-inactivated fetal bovine serum (Gibco, Waltham, MA, USA). All cells were kept at 37 °C in a humidified atmosphere of 5% CO_2_. Drug treatments were performed directly on cultured media. Bafilomycin A1 (BafA1; 50 nM), GA-017 (10 μM), and Forskolin (10 μM) were all purchased from GLPBIO (Montclair, America).

### Lentivirus construction and transfection

The lentiviruses used in this study were constructed and supplied by Genomeditech Co., Ltd. (Shanghai, China). The shRNA sequences were specifically designed to target human *rgs20*. The three shRNA sequences used are listed in Supplementary Table 1. Following the manufacturer’s instructions, lentiviruses were transfected into the cells using the transfection reagent for 48 h. Transfection efficiency was assessed by western blot analysis.

### Immunoblotting

Proteins were extracted and detected as previously described [16]. Briefly, SDS-PAGE (Epizyme, Shanghai, China) was used to separate proteins and then transferred onto PVDF membranes (Millipore, Germany). Membrane blocking was performed by using a 5% dry milk solution dissolved in Tris Buffer Saline with 1% Tween 20 (TBST) and incubated overnight at 4 °C with the primary antibody. A secondary antibody was used, and immunoblots were developed using an ECL western substrate (Epizyme, Shanghai, China). The bands were visualized using a gel imaging system (Tanon 5200, Shanghai, China), and ImageJ software was used to quantify the integrated optical density of the bands. The antibodies used in this study are listed in Supplementary Table 2.

### CCK-8 assay

Cells were seeded in 96-well plates at a density of 1,000 cells per well and assessed for proliferation using Cell Counting Kit-8 (CCK-8) (GK10001, Biosharp, Beijing, China). After incubation for 24, 48, 72, and 96 h, the culture medium was replaced with 100 μl of 10% CCK-8 serum-free medium. Following an additional hour in a cell incubator, the absorbance of cells was measured at 450 nm.

### Tumor colony formation assay

A single-cell suspension containing 5 × 10^3^ cells was cultured in RPMI Medium 1640 (10% FBS) for 10 days in a six-well plate. Once most of the cell clones exceeded the 50-cell mark, the cells were washed twice with PBS, treated with 4% paraformaldehyde for 10 min, and stained with 0.05% crystal violet for 15 min at room temperature. After rinsing off the stain, the number of colonies (50 cells/colony) was counted under a microscope at 40× magnification (Olympus, Japan).

### Transcriptome sequencing

TRIzol reagent was employed to extract total RNA from both the control group and the RGS20-OE group of lung adenocarcinoma H1299 cells, with three replicates per group. The transcriptome sequencing and subsequent analysis of the sequencing results were outsourced to OE Biotech Co. Ltd. in Shanghai, China. The libraries were subjected to sequencing on an Illumina HiSeq X Ten platform, generating 150 bp paired-end reads for subsequent analysis of differentially expressed genes.

### Immunofluorescence

Cells were initially seeded on 12 mm coverslips in 24-well plates. After 24 h of culture, cells underwent fixation with 4% paraformaldehyde and were permeabilized in 0.1% Triton X-100/PBS. Subsequently, cells were blocked for 2 h in 5% bovine serum albumin/PBS, followed by overnight incubation at 4 °C with primary antibody at a dilution of 1:100. Afterward, fluorescence-labeled secondary antibodies were applied for 2 h. Nuclei were strained using DAPI. Imaging was performed using a confocal laser scanning microscope at 400× magnification (Leica STELLARIS 5, Germany). The antibodies used in this study are listed in Supplementary Table 2.

### Xenograft nude mice model

Four weeks old female BALB/c nude mice were purchased from the Beijing Vital River Laboratory (Beijing, China), bred, and housed in a specific pathogen-free (SPF) environment. All animal experiments adhered to the ethical guidelines of the Institutional Ethics Committee of Qingdao University, China (Ethical approval number: 20221117BALB/cnude2420221230061). The antibodies used in this study are listed in Supplementary Table 2.

### Statistical analysis

The data analysis was performed using Prism 5.0 (Graph Pad Software, Inc., La Jolla, CA, USA). A student’s T-test or one-way ANOVA, was employed to analyze differences between groups, including the expression of clinicopathological parameters and immunohistochemical indices. The results are presented as the mean ± SD of three or more observations in each experiment, and a significance level of *p* < 0.05 was considered statistically significant.

## Results

### Elevated expression of RGS20 is associated with a poor prognosis of NSCLC

**Fig. 1:**
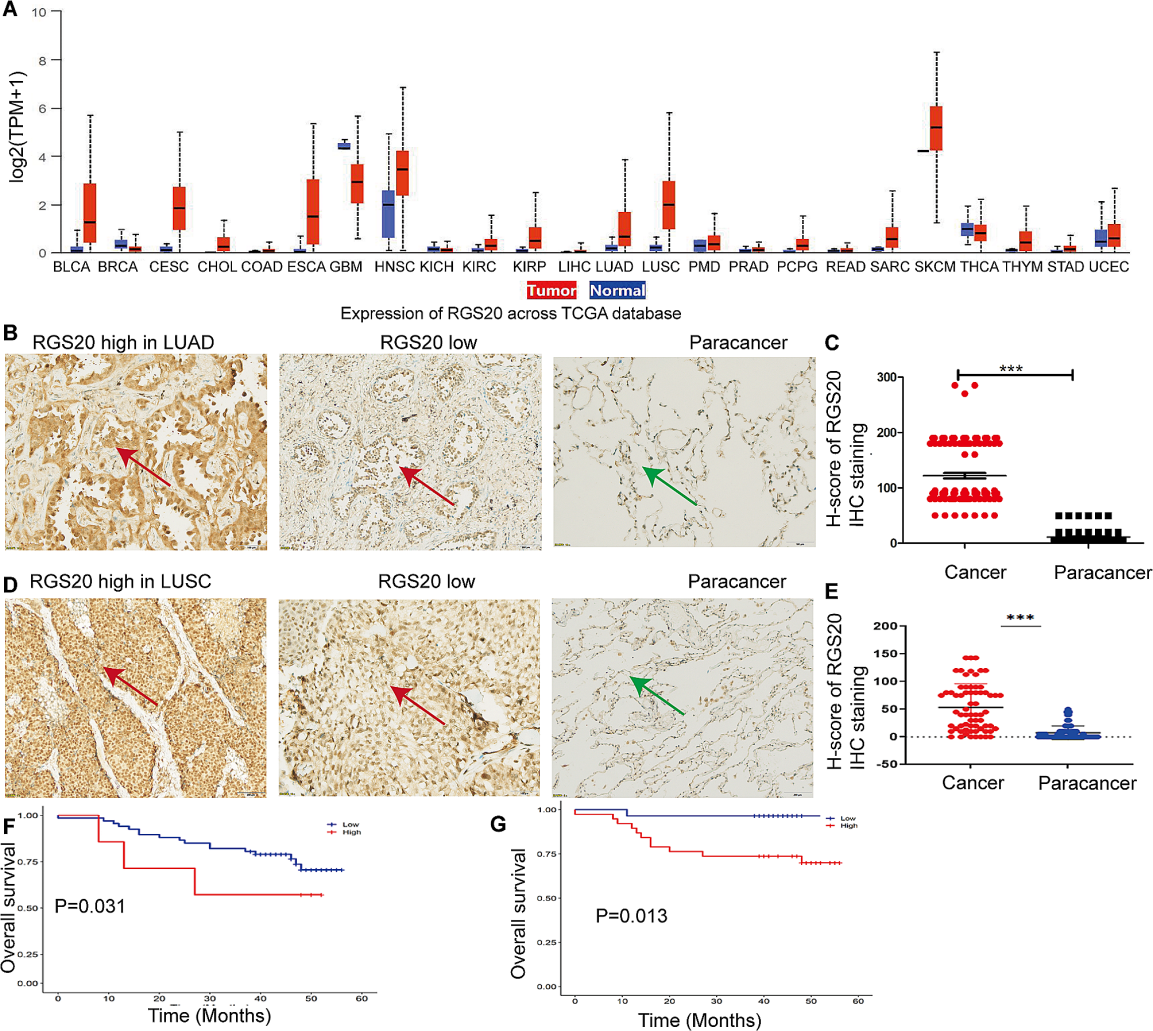
Elevated expression of RGS20 is associated with a poor prognosis for NSCLC **A** The mRNA expression of rgs20 in many cancers was assessed using TCGA database. **B** The representative IHC staining of the RGS20 protein in lung adenocarcinoma tumors (red arrow) and adjacent non-cancerous tissues (green arrow). **C** The quantitative analysis of IHC staining results for RGS20 in 126 LUAD tissues. **D** The representative IHC staining of RGS20 protein in lung squamous carcinoma tissue microarray (red arrow) and adjacent non-cancerous tissues (green arrow). **E** The quantitative analysis of IHC staining results for RGS20 in 75 LUSC tissues. Kaplan-Meier analysis of the association between RGS20 protein levels and overall survival in 126 LUAD (**F**) and 75 LUSC (**G**) patients. The results in C and E were quantified with CaseViewer (version 2.3) and presented as H-Scores. The samples from patients were divided into high and low subgroups based on the median H-score of IHC staining, which represents the expression level of RGS20. * *p* < 0.05, **p < 0.01, ***p < 0.001

We investigated *rgs20* expression profiles across multiple tumors using the Cancer Genome Atlas (TCGA) database (https://ualcan.path.uab.edu/cgi-bin/Pan-cancer.pl?genenam=RGS20). Elevated rgs20 expression was noticed in lung adenocarcinoma (LUAD), lung squamous cell carcinoma (LUSC), and other tumors (Fig. 1A). Specifically, RGS20 was more highly expressed in adenocarcinomas than in adjacent noncancerous tissues (Fig. 1B and C) and exhibited markedly higher expression in squamous lung tissue than that of normal lung tissue (Fig. 1D and E). Furthermore, a positive correlation was observed between RGS20 expression levels and the T stage of the tumor as well as cervical lymph node metastasis (N stage) (Table 1). Kaplan-Meier analysis demonstrated that patients with high RGS20 expression levels exhibited significantly shorter five-year overall survival rates in both LUAD (Fig. 1F) and LUSC (Fig. 1G). Multivariate Cox regression analysis indicated that RGS20 expression was an independent prognostic factor for LUSC (Table 2). Taken together, these findings suggest RGS20 as a potential biomarker for NSCLC diagnosis, with elevated expression levels positively correlated with a poor prognosis.

**Table 1:** Correlation between RGS20 expression and clinicopathological characteristics in lung adenocarcinoma

	variables	RGS20 expression		total	χ^2^	*p* value
		low	high			
Age (year)					0.152	0.697
	≤ 60	37	25	60		
	> 60	36	28	66		
Sex					0.021	0.886
	male	28	21	49		
	Female	45	32	77		
T stage					5.690	0.029^*^
	T1-T2	63	39	102		
	T3/T4	10	14	24		
N stage					10.242	0.002^*^
	N0	74	34	108		
	Nx-N2	8	10	18		
TNM stage					2.799	0.175
	I-II	71	45	116		
	III	5	6	11		
Tumor rize					0.833	0.361
	≤ 2 cm	50	37	87		
	> 2 cm	19	20	39		

* Statistically significant (*p* < 0.05)

**Table 2:** Univariate and multivariate analyses of the factors correlated with Overall survival of LUSC patients Variables in the Equation

variables	Univariate analysis					Multivariate analysis			
	*p* value	HR	95%CI			*p* value	HR	95%CI	
			Lower limit	Upper limit				Lower limit	Upper limit
expression	0.040	8.573	1.104	66.549		0.018^*^	12.017	1.526	94.617
Age	0.058	2.987	0.962	9.270					
sex	0.494	0.043	0.000	346.179					
Tumor size	0.496	0.634	0.170	2.357					
Grade stage	0.189	0.029	0.000	5.700					
TNM stage	0.173	2.195	0.708	6.808					
T stage	0.013	4.214	1.355	13.100		0.002^*^	6.061	1.929	19.041
N stage	0.344	1.728	0.557	5.357					

LUSC: lung squamous cell carcinoma, HR: Hazard Ratio, CI: Confidence Interval,

* Statistically significant (*p* < 0.05)

### RGS20 promotes NSCLC cell proliferation by activating autophagy in vitro

**Fig. 2:**
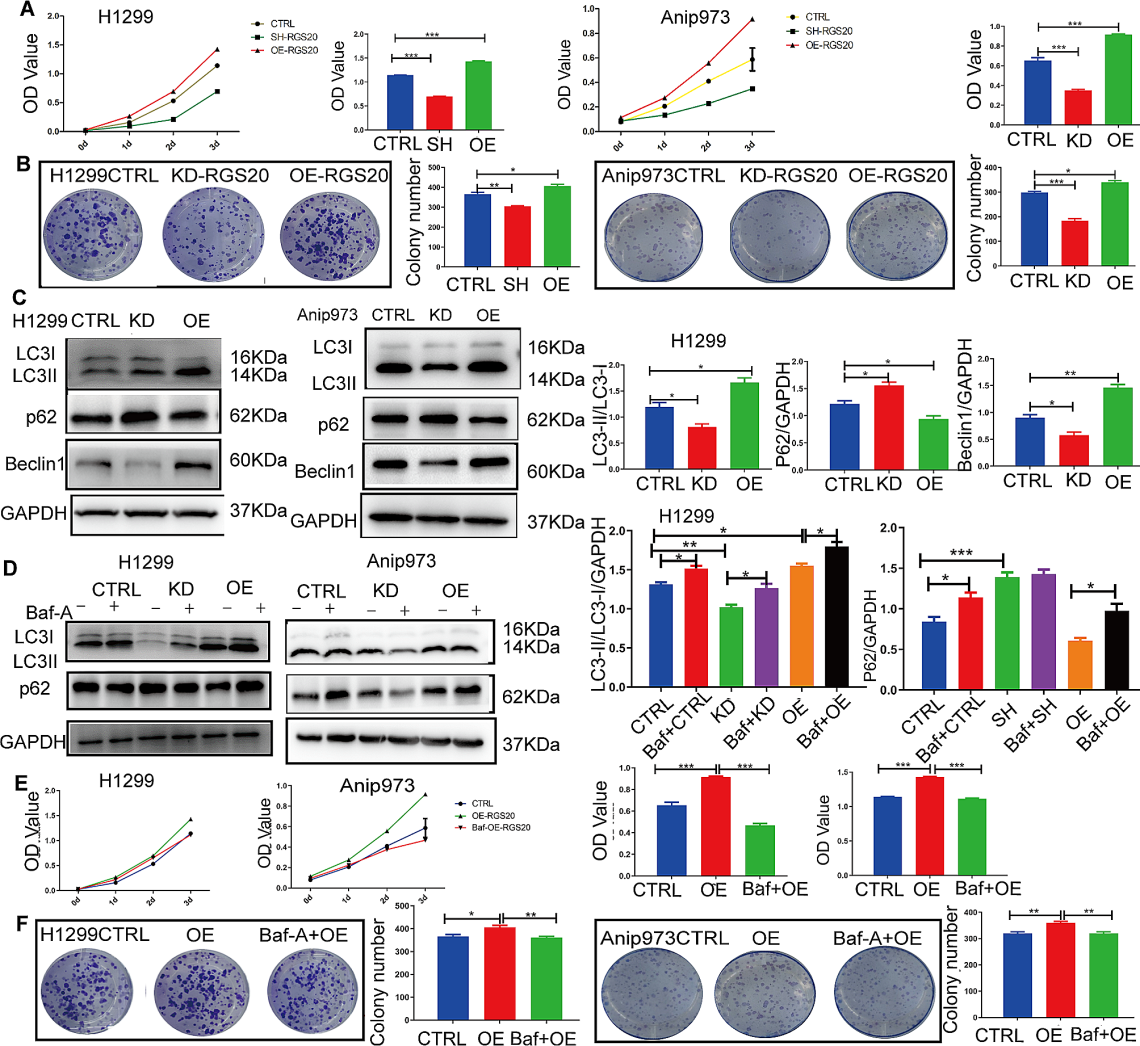
RGS20 promotes NSCLC cell proliferation and activates autophagy in vitro **A** Cell proliferation of three different groups (CTRL, RGS20-KD, and RGS20-OE) in H1299 and Anip973 cells was detected by the Cell Counting Kit-8 (CCK-8) assay. **B** Colony formation assays show the colony-forming ability of H1299 and Anip973 cells in three groups (CTRL, RGS20-KD, and RGS20-OE). The number of colonies was counted and is displayed on the right side. **C** Representative western blot results show the protein levels of autophagy-related proteins and the corresponding quantification analysis. **D** Representative western blot results show the protein levels of autophagy related proteins after blockage of autophagy flux by Baf-A1 (50 nM, 2 h) in three groups (CTRL, RGS20-KD, and RGS20-OE) of H1299 and Anip973 cells. The corresponding quantification analysis results were listed on the right. **E** CCK-8 assay was performed to assess the proliferation of H1299 and Anip973 cells in the RGS20-OE group treated with Baf-A1 (50 nM, 2 h). **F** Colony formation assays were used to analyze the colony-forming ability of H1299 and Anip973 cells in the RGS20-OE group treated with Baf-A1 (50 nM, 2 h). The number of colonies was counted and is displayed on the right side. * *p* < 0.05, **p < 0.01, ***p < 0.001

The role of RGS20 in NSCLC was further examined using H1299 and Anip973 cells (Supplementary Fig. 1A). Next, RGS20 overexpression (RGS20-OE) and knockdown (RGS20-KD) cell lines were generated using lentiviral vectors (Supplementary Fig. 1B, C, D). RGS20 enhanced cell proliferation and foci formation (Fig. 2A and B), consistent with previous reports [11]. Since autophagy plays a critical role in various stages of tumor progression [15], we investigated whether RGS20 promotes cell proliferation by increasing autophagy in NSCLC cells. RGS20 overexpression increased the ratio of LC3II/LC3I protein levels, elevated BECLIN1 expression, and reduced P62 protein levels, indicating increased autophagy (Fig. 2C). To further investigate the potential link between autophagy and proliferation in RGS20-OE cells, Bafilomycin A1 (Baf-A1) was used to block autophagic flux. The observed accumulation of LC3-II and P62 suggested that Baf-A1 effectively blocked the autophagic flux (Fig. 2D). Notably, suppression of autophagic flux resulted in a decrease in the proliferation of RGS20-OE cells (Fig. 2E and F). Taken together, these findings suggest that RGS20 induces NSCLC cell proliferation by upregulating autophagy in vitro.

### RGS20 inhibites the Hippo signaling pathway in NSCLC

**Fig. 3:**
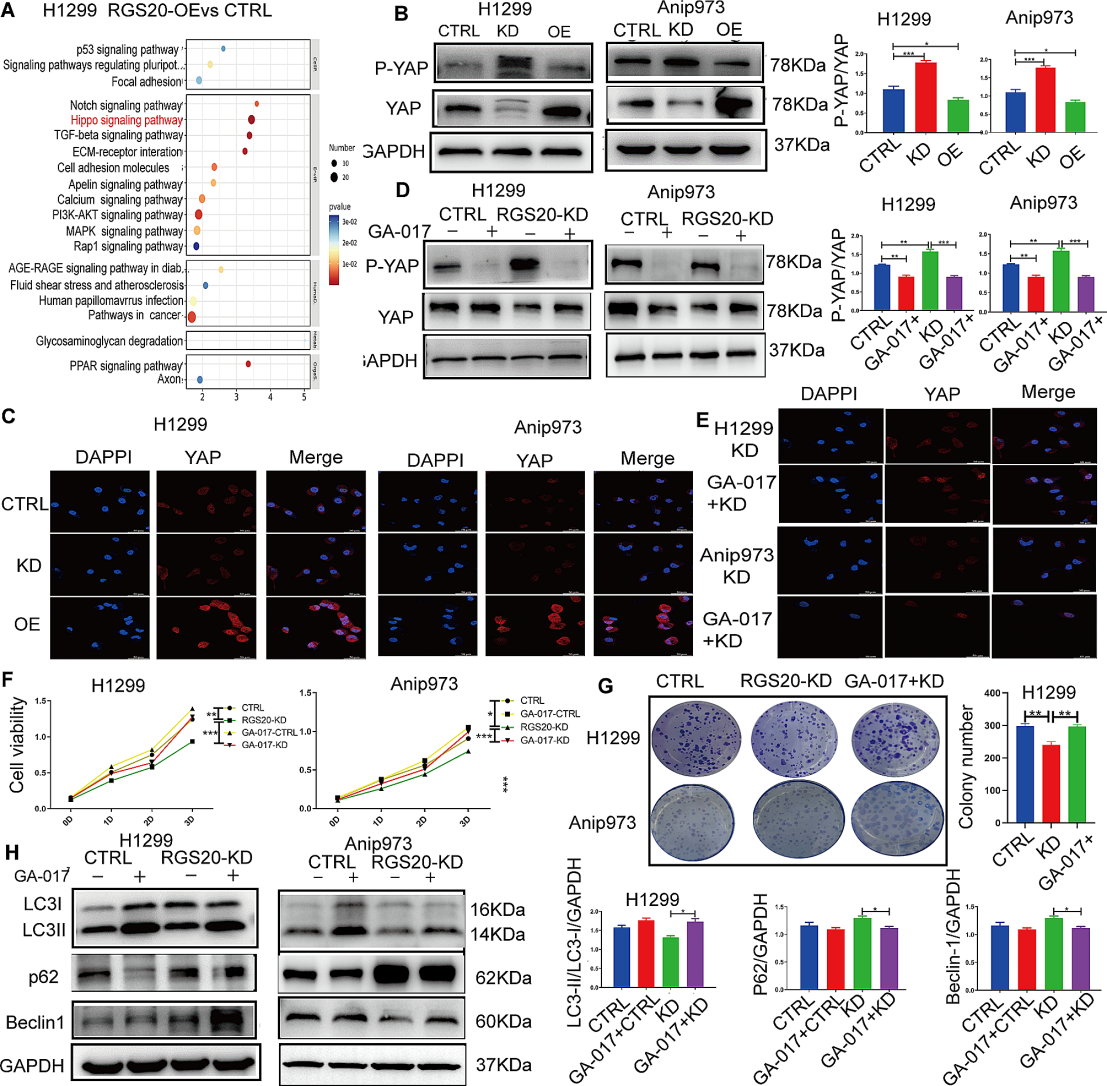
RGS20 inhibits the Hippo signaling pathway in NSCLC **A** KEGG pathway analysis was performed by transcriptome sequencing to identify the signaling pathway in which differentially expressed genes were enriched. **B** The representative Western blot result shows the protein levels of p-YAP and YAP in the control, RGS20-KD, and RGS20-OE groups of H1299 and Anip973 cells. The corresponding quantification analysis results are shown on the right. **C** Representative immunofluorescence images show the nuclear translocation of YAP in three different groups of H1299 and Anip973 cells. **D** The representative Western blot result shows the p-YAP and YAP protein levels after the blockage of the Hippo signaling pathway with GA-017 (10 μM, 24 h) in the RGS20-KD and control groups. The corresponding quantification analysis results are shown on the right. **E** Representative immunofluorescence images of YAP in the RGS20-KD group after treatment with GA-017. **F** CCK-8 assay to detect the proliferation of H1299 and Anip973 cells after treatment with GA-017 (10 μM, 72 h) in the control and RGS20-KD groups. **G** Colony formation assays were used to analyze the colony-forming ability of H1299 and Anip973 cells treated with GA-017 (10 μM, 72 h). **H** Representative Western blot result shows autophagy-related protein levels after the blockage of the Hippo signaling pathway with GA-017 (10 μM, 24 h) in the RGS20-KD and control groups. * *p* < 0.05, **p < 0.01, ***p < 0.001

Transcriptome sequencing was performed to explore the specific molecular mechanisms of action of RGS20 in NSCLC. The Kyoto Encyclopedia of Genes and Genomes (KEGG) pathway enrichment analysis revealed 20 signaling pathways. Notably, differentially expressed genes were predominantly enriched in the Hippo signaling pathway (Fig. 3A). Therefore, we hypothesized that RGS20 promotes NSCLC cell proliferation and autophagy via the Hippo signaling pathway. Indeed, phosphorylated YAP (p-YAP) levels were decreased in RGS20-OE cells and increased in RGS20-KD cells (Fig. 3B). Furthermore, RGS20-KD cells exhibited reduced YAP nuclear translocation, whereas RGS20-OE cells demonstrated increased nuclear translocation of YAP (Fig. 3C). To examine the potential association between RGS20 and the Hippo signaling pathway in NSCLC, RGS20-KD cells were treated with GA-017, a specific inhibitor of the Hippo signaling pathway. GA-017 treatment led to a decrease in p-YAP protein levels (Fig. 3D) and an increase in nuclear translocation of YAP (Fig. 3E), confirming the inhibition of the Hippo signaling pathway in RGS20-KD cells. In addition, CCK8 and clone formation assays revealed accelerated cell proliferation and foci formation in RGS20-KD cells treated with GA-017 (Fig. 3F and G). These findings suggest that inhibition of the Hippo signaling pathway in RGS20-KD cells promotes cell growth and clonogenic potential. Moreover, the levels of autophagy-related proteins, such as LC3-II/LC3-I and BECLIN-1, were upregulated in RGS20-KD cells following inhibition of the Hippo signaling pathway (Fig. 3H). These results suggest that RGS20 exerts an inhibitory effect on the Hippo signaling pathway in NSCLC, potentially affecting autophagy regulation.

### RGS20 promotes cell proliferation and enhances autophagy by suppressing the PKA-Hippo signaling pathway in NSCLC

**Fig. 4:**
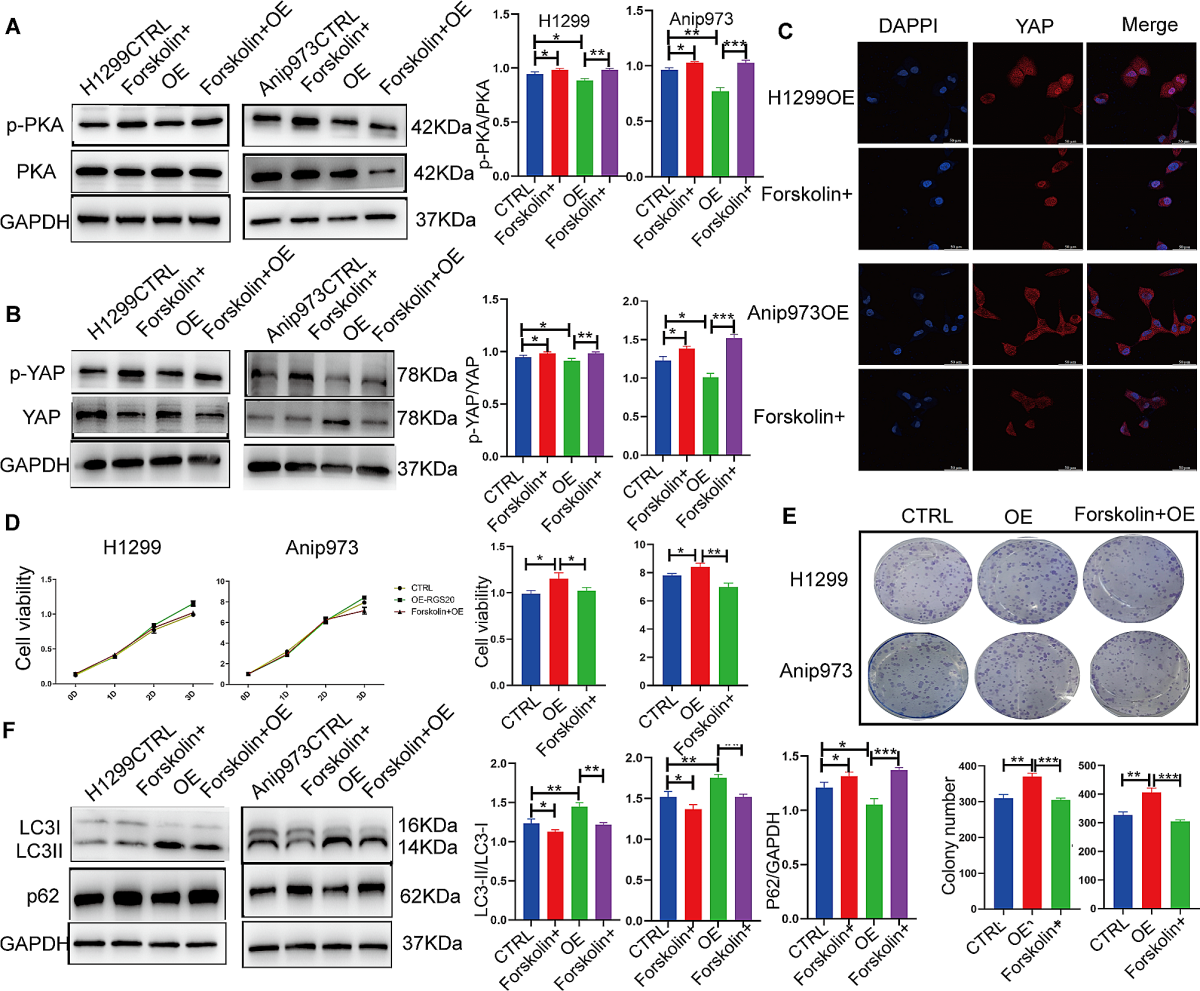
RGS20 promotes cell proliferation and enhances autophagy by inhibiting the PKA-Hippo signaling pathway in vitro **A** Representative western blot result shows the protein level of p-PKA and PKA in RGS20-OE cells after treatment with forskolin (10 μM, 72 h). The corresponding results of the quantification analysis are displayed on the right. **B** Representative western blot result shows the protein level of p-YAP and YAP after the activation of the PKA signaling pathway with forskolin (10 μM, 72 h) in RGS20-OE and control cells. **C** Representative immunofluorescence images of YAP in the RGS20-OE group after treatment with forskolin. **D** The CCK-8 assay detects the proliferation of H1299 and Anip973 cells after treatment with forskolin (10 μM, 72 h) in the RGS20-OE and control groups. **E** Colony formation assays were used to analyze the colony-forming ability of H1299 and Anip973 cells after treatment with forskolin (10 μM, 72 h). **F** Representative western blot result shows the protein levels of autophagy-related proteins after the activation of the PKA signaling pathway with forskolin (10 μM, 72 h) in the RGS20-OE and control groups. * *p* < 0.05, **p < 0.01, ***p < 0.001

Because GPCRs represent a significant signaling branch upstream of the Hippo pathway [16], RGS proteins may modulate cell proliferation and autophagy by regulating the PKA-mediated GPCR signaling pathway. Consistently, the phosphorylation of PKA was reduced in RGS20-OE cells and increased in RGS20-KD cells (Fig. 4A). To investigate the relationship between PKA and the Hippo signaling pathway, RGS20-OE cells were treated with forskolin, a cyclic adenosine monophosphate (cAMP) activator. Subsequently, an elevated level of p-YAP compared to that of total YAP (Fig. 4B) and a decrease in the translocation of YAP to the nucleus (Fig. 4C) were observed. In addition, CCK-8 and clone formation assays revealed decreased cell proliferation and foci formation in RGS20-OE cells following forskolin administration (Fig. 4D and E). Furthermore, the LC3-II/LC3-I ratio decreased in RGS20-OE cells treated with forskolin, indicating a reduction in autophagy. Meanwhile, the protein level of P62 was increased, suggesting impaired autophagy (Fig. 4F). These findings demonstrate that RGS20 promotes cell proliferation and enhances autophagy by suppressing the PKA-Hippo signaling pathway in NSCLC.

### RGS20 enhances the tumorigenesis of NSCLC cells in vivo

**Fig. 5:**
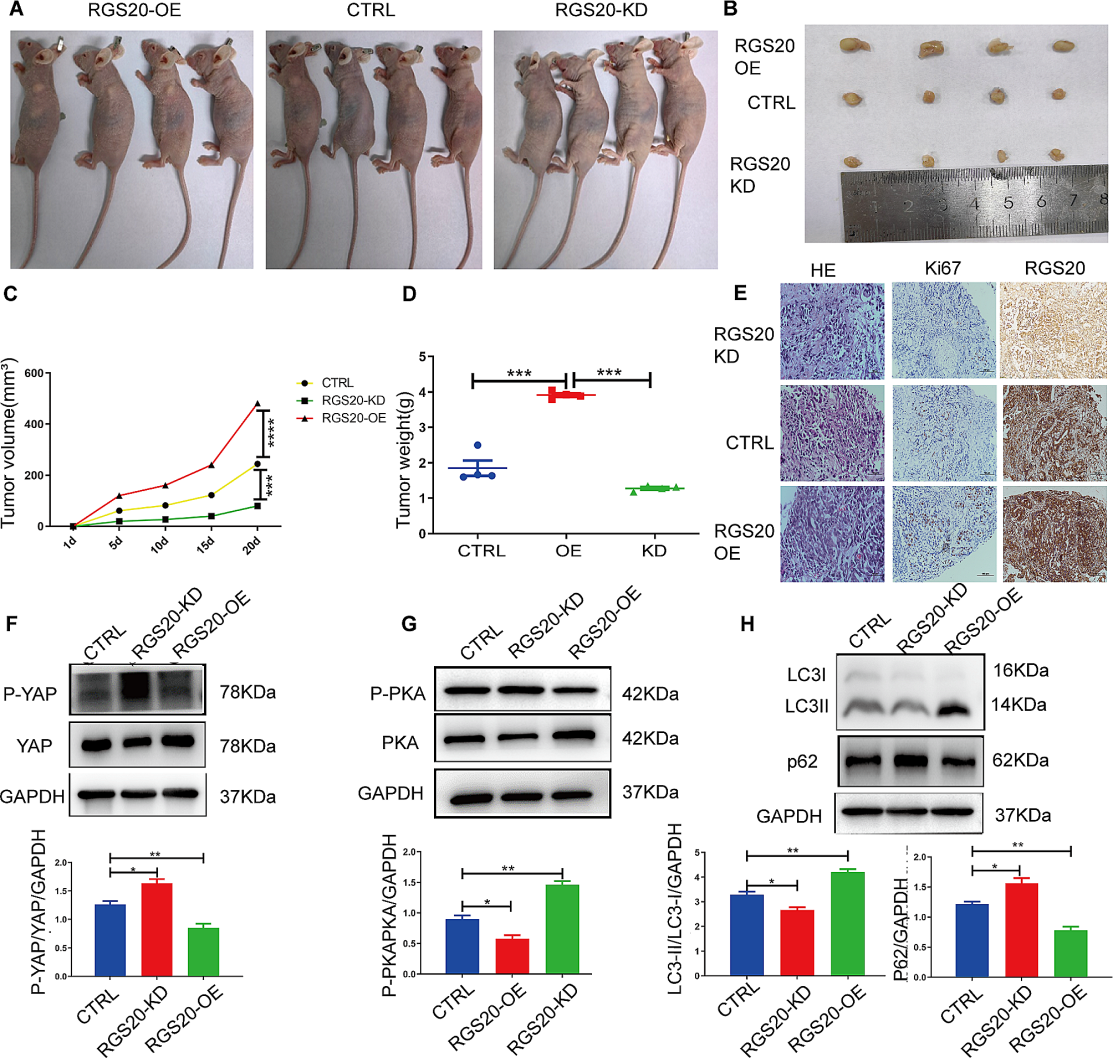
RGS20 enhances the tumorigenesis of NSCLC cells in vivo **A** Tumor-promoting effects of RGS20 in a xenograft mouse model. The representative images of tumor-bearing mice that were photographed at the end of the third week of experimentation. **B** Representative images of the dissected tumors. A ruler was used to indicate the size of the tumors. **C** The tumor growth curve was plotted using xenograft tumor volume data. **D** Tumor weight was measured after tumor excision. **E** Representative images of HE staining and IHC staining of Ki-67 and RGS20. Representative western blot results show the protein level of the PKA-Hippo (**F, G**) signaling pathway and autophagy-related proteins **(H).** The corresponding quantification analysis results are listed below. Protein levels were normalized to GAPDH. Results are presented as the mean ± SD (*n* = 3). * *p* < 0.05, **p < 0.01, ***p < 0.001

To further assess the tumorigenic effects of RGS20 on NSCLC cells in vivo, we established a xenograft nude mouse model. The growth curve of xenograft tumors showed that RGS20 promoted tumorigenesis in vivo (Fig. 5A and B). Xenograft tumors in the RGS20-OE group were larger and heavier than those in the control group. We observed significant inhibition of tumor growth in the RGS20-KD group (Fig. 5C and D). Hematoxylin and eosin (HE) staining showed that all tumors were solid, with a higher Ki-67 index in tumors of the RGS20-OE group than in those of the control group (Fig. 5E). To validate the mechanisms by which RGS20 promotes the proliferation of NSCLC cells in vivo, we measured protein levels linked to the PKA-Hippo signaling pathway and autophagy in solid tumors. We noticed a significant decrease in the ratio of p-PKA/PKA and p-YAP/YAP protein levels in the tumor tissues of the RGS20-OE group compared to that of the control group (Fig. 5F and G). Further, the ratio of LC3-II/LC3-I protein levels significantly rose in the tumors of the RGS20-OE group compared to that in the control group, whereas P62 protein levels showed the opposite trend (Fig. 5H). Therefore, RGS20 enhanced the tumorigenesis of NSCLC cells in vivo by increasing autophagy via the suppression of the PKA-Hippo signaling pathway.

## Discussion

In this study, we observed elevated RGS20 expression in NSCLC tumors, correlating with a poor prognosis. Additionally, high RGS20 levels were significantly related to advanced T stage, lymphatic metastasis, and poorer 5-year overall survival rates. Multivariate Cox regression analysis confirmed RGS20 expression as an independent prognostic factor. These findings suggest that RGS20 is likely an oncogene and can serve as a novel diagnostic and prognostic marker for NSCLC.

RGS proteins negatively regulate G-protein signaling by binding to G-protein α subunits and facilitating GTP hydrolysis, thereby dampening signal transduction [17]. A previous study assessing the global map of GPCR by RGS proteins reported that RGS A/RZ subfamily members (RGS17, RGS19, and RGS20) uniquely regulate Gαz, Gαi/o, and Gαq proteins [18]. RGS17 has been reported to induce lung and prostate cancer proliferation via the cAMP-PKA-CREB pathway [19]. Though a few other studies have explored the relationship between the cAMP-PKA pathway and pathogenic lung inflammation [20] or injury [21], limited research exists on the role of this pathway in NSCLC. Though a few exceptions exist, such as a study that revealed that endogenous glutamate regulates ferroptosis sensitivity in LUAD through PKA signaling [22], a second study demonstrated the involvement of MHY4571 in the regulation of cAMP-PKA signaling in squamous cell lung cancer treatment [23]. Our study highlighted RGS20’s inhibition of cAMP-PKA signaling, which was reversed by forskolin in RGS20-OE NSCLC cells.

As a downstream branch of GPCR signaling, the Hippo pathway plays a crucial role in regulating cell survival [16], proliferation [24], differentiation and organ size [25, 26]. The core Hippo signaling pathway in mammals includes mammalian sterile 20-like kinase 1/2 (MST1/2), large tumor suppressor kinase 1/2 (LATS1/2), and YAP/transcriptional coactivator with a PDZ-binding motif (TAZ) [27]. Phosphorylation of YAP/TAZ leads to its cytoplasmic retention and ubiquitination-dependent proteasomal degradation. When upstream kinases are inactivated, dephosphorylated YAP/TAZ translocates to the nucleus and induces the expression of target genes [16, 27]. Elevated expression and nuclear localization of YAP have been observed in many cancers, including cervical squamous cell carcinoma, colorectal cancer, and esophageal carcinoma [28–30] and have also been associated with poor prognosis in esophageal carcinoma and ovarian cancer [30, 31]. Elevated YAP levels have also been detected in lung cancer patients with acquired EGFR inhibitor resistance [32] and TAZ level was a prognostic factor in NSCLC progression [33, 34]. Our study demonstrated that overexpression of RGS20 decreased the expression of p-YAP and increased YAP translocation to the nucleus, which further verified the important correlation between GPCR and the Hippo pathway.

The Hippo signaling pathway also acts as an upstream regulator of autophagy [35, 36]. Autophagy plays a dual role in tumors, acting as a double-edged sword by both promoting tumor growth and inhibiting tumor progression. For example, loss of autophagy results in the incidence of hepatocellular carcinoma [37]. Further, autophagy has been reported to suppress breast cancer metastasis by degrading the neighbor of BRCA1 gene 1 (NBR1) [38, 39]. However, autophagy can also promote pancreatic cancer [40]. The potential ability of autophagy to modulate cell death and regulate autophagy has made it an effective interventional strategy for cancer therapy [41–43]. Lung cancer has high basal autophagic activity, which is associated with autophagy during tumorigenesis [44]. A recent study revealed that sex-determining region Y-box 2 (SOX2) promotes LC3A expression and enhances the proliferation of lung cancer cells. Autophagy is activated in serine/threonine kinase 11 (STK11) mutant lung cancer [45]. Our study found that RGS20 overexpression enhanced autophagy by increasing the expression of the proteins LC3 II and BECLIN1 while decreasing the protein level of P62. Further, our in vitro experiments demonstrated that the viability of RGS20-OE cells was decreased by blocking autophagy. Furthermore, we noticed that the tumor-promoting activity of RGS20 relies on autophagy. Our findings, along with previous research, point to the crucial role played by autophagy in various cancer types. However, the specific molecular mechanisms by which RGS20 influences autophagy remain unclear and require further exploration by future studies.

**Fig. 6:**
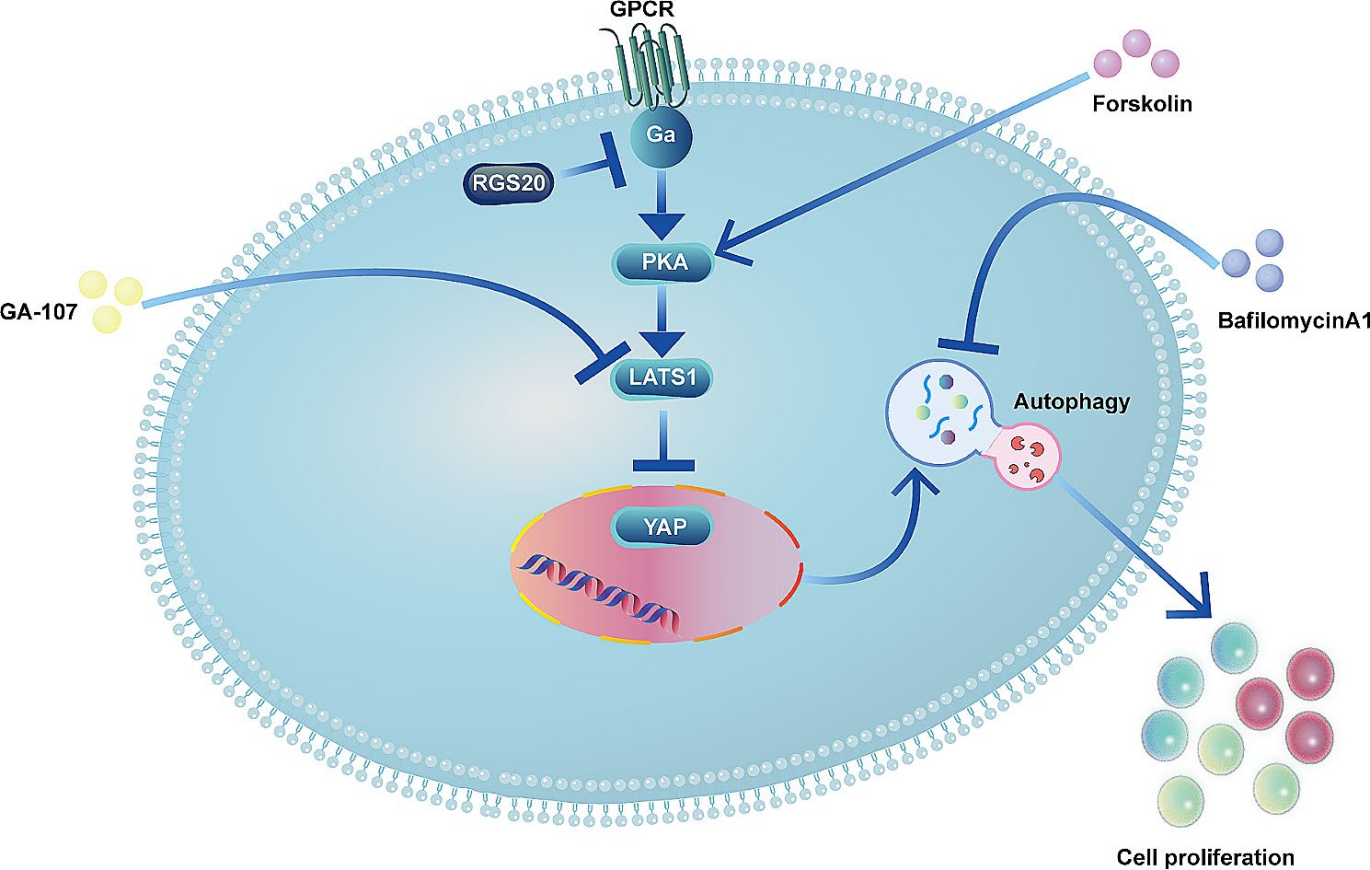
Graphical Abstract RGS20 promotes NSCLC proliferation and activates autophagy by inhibiting the PKA-Hippo signaling pathway

In conclusion, our study suggests that elevated RGS20 expression in NSCLC is associated with a poor five-year survival rate. RGS20 promotes NSCLC cell proliferation by enhancing autophagy via suppression of the PKA-Hippo signaling pathway (Fig. 6). Further research on RGS20 may offer novel and promising therapeutic targets, particularly for the treatment of patients with drug-resistant NSCLC.

However, there are still gaps in our understanding of the role of RGS20 in NSCLC. For example, the specific mechanisms by which RGS20 activates autophagy, its impact on other tumor behaviors such as invasion, metastasis, and metabolism, and the existence of small molecule inhibitors targeting RGS20, all require further experimental investigations to be addressed in future.

### Electronic supplementary material

Below is the link to the electronic supplementary material.

Supplementary Material 1

Supplementary Material 2

Supplementary Material 3

Supplementary Material 4

Supplementary Material 5

## Data Availability

The data that support the findings of this study are available from the corresponding author upon reasonable request.

## Abbreviations

BafA1: Bafilomycin A1
CCK-8: Cell counting kit-8
DAPI: 4’,6-diamidino-2-phenylindole
ECM: Extracellular matrix
GO: Gene ontology
GPCR: G-protein-coupled receptors
H&E: Hematoxylin and eosin
Hippo: Hippopotamus
IHC: Immunohistochemistry
KEGG: Kyoto encyclopedia of genes and genomes
LUAD: Lung adenocarcinoma
LUSC: Lung squamous cell carcinoma
NSCLC: Non-small cell lung cancer
RGS: Regulators of G protein signaling
RGS20: Regulator of G protein signaling 20
RT-qPCR: Reverse-transcription quantitative real-time polymerase chain reaction
shRNAs: Short hairpin RNAs
SPF: Specific pathogen-free
TAZ: Transcriptional coactivator with PDZ-binding motif
TBST: Tris-buffered saline tween
TEAD: Transcriptional enhanced associate domain
TGF-β: Transforming growth factor-β
YAP: Yes-associated protein

## References

[1] Chen Z, Fillmore CM, Hammerman PS, Kim CF, Wong KK (2014). Non-small-cell lung cancers: a heterogeneous set of diseases. Nat Rev Cancer.

[2] Siegel RL, Miller KD, Jemal A (2016). Cancer statistics, 2016. CA Cancer J Clin.

[3] Guo H, Zhang J, Qin C, Yan H, Liu T, Hu H et al. Biomarker-targeted therapies in non-small cell lung cancer: current status and perspectives. Cells. 2022;11(20). 10.3390/cells11203200 PMC9600447 36291069

[4] Chen R, Manochakian R, James L, Azzouqa AG, Shi H, Zhang Y (2020). Emerging therapeutic agents for advanced non-small cell lung cancer. J Hematol Oncol.

[5] Wang M, Herbst RS, Boshoff C (2021). Toward personalized treatment approaches for non-small-cell lung cancer. Nat Med.

[6] Hsu WH, Yang JC, Mok TS, Loong HH (2018). Overview of current systemic management of EGFR-mutant NSCLC. Annals Oncology: Official J Eur Soc Med Oncol.

[7] Rotow J, Bivona TG (2017). Understanding and targeting resistance mechanisms in NSCLC. Nat Rev Cancer.

[8] Alqinyah M, Hooks SB (2018). Regulating the regulators: epigenetic, transcriptional, and post-translational regulation of RGS proteins. Cell Signal.

[9] Shi D, Tong S, Han H, Hu X (2022). RGS20 promotes Tumor Progression through modulating PI3K/AKT signaling activation in Penile Cancer. J Oncol.

[10] Yang L, Lee MM, Leung MM, Wong YH (2016). Regulator of G protein signaling 20 enhances cancer cell aggregation, migration, invasion and adhesion. Cell Signal.

[11] Li G, Wang M, Ren L, Li H, Liu Q, Ouyang Y (2019). Regulator of G protein signaling 20 promotes proliferation and migration in bladder cancer via NF-kappaB signaling. Biomed Pharmacother.

[12] Huang G, He X, Wei XL (2018). lncRNA NEAT1 promotes cell proliferation and invasion by regulating miR–365/RGS20 in oral squamous cell carcinoma. Oncol Rep.

[13] Li Q, Jin W, Cai Y, Yang F, Chen E, Ye D (2017). Regulator of G protein signaling 20 correlates with clinicopathological features and prognosis in triple-negative breast cancer. Biochem Biophys Res Commun.

[14] Jiang L, Shen J, Zhang N, He Y, Wan Z (2021). Association of RGS20 expression with the progression and prognosis of renal cell carcinoma. Oncol Lett.

[15] Li X, He S, Ma B (2020). Autophagy and autophagy-related proteins in cancer. Mol Cancer.

[16] Luo J, Yu FX. GPCR-Hippo signaling in cancer. Cells. 2019;8(5). 10.3390/cells8050426 PMC6563442 31072060

[17] Watson N, Linder ME, Druey KM, Kehrl JH, Blumer KJ (1996). RGS family members: GTPase-activating proteins for heterotrimeric G-protein alpha-subunits. Nature.

[18] Masuho I, Balaji S, Muntean BS, Skamangas NK, Chavali S, Tesmer JJG (2020). A Global Map of G protein signaling regulation by RGS proteins. Cell.

[19] Bodle CR, Mackie DI, Roman DL (2013). RGS17: an emerging therapeutic target for lung and prostate cancers. Future Med Chem.

[20] Bae GH, Kim YS, Park JY, Lee M, Lee SK, Kim JC (2022). Unique characteristics of lung-resident neutrophils are maintained by PGE2/PKA/Tgm2-mediated signaling. Blood.

[21] Yang X, Ma X, Don O, Song Y, Chen X, Liu J (2020). Mesenchymal stem cells combined with liraglutide relieve acute lung injury through apoptotic signaling restrained by PKA/β-catenin. Stem Cell Res Ther.

[22] Zhang X, Yu K, Ma L, Qian Z, Tian X, Miao Y (2021). Endogenous glutamate determines ferroptosis sensitivity via ADCY10-dependent YAP suppression in lung adenocarcinoma. Theranostics.

[23] Chung JH, Choi HJ, Kang YJ, Kim YS, Lee SY, Kwon RJ (2022). MHY4571, a novel diarylcyclohexanone derivative, exerts anti-cancer activity by regulating the PKA-cAMP-response element-binding protein pathway in squamous cell lung cancer. Experimental Hematol Oncol.

[24] Dey A, Varelas X, Guan KL (2020). Targeting the Hippo pathway in cancer, fibrosis, wound healing and regenerative medicine. Nat Rev Drug Discovery.

[25] Driskill JH, Pan D (2021). The Hippo Pathway in Liver Homeostasis and Pathophysiology. Annu Rev Pathol.

[26] Fu M, Hu Y, Lan T, Guan KL, Luo T, Luo M (2022). The Hippo signalling pathway and its implications in human health and diseases. Signal Transduct Target Therapy.

[27] Ma S, Meng Z, Chen R, Guan KL (2019). The Hippo Pathway: Biology and Pathophysiology. Annu Rev Biochem.

[28] Cancer Genome Atlas Research N, Albert Einstein College of M, Analytical Biological S, Barretos Cancer H, Baylor College of M, Beckman Research Institute of City of H, et al. Integrated genomic and molecular characterization of cervical cancer. Nature. 2017;543(7645):378–84. 10.1038/nature21386 PMC5354998 28112728

[29] Wang Y, Xu X, Maglic D, Dill MT, Mojumdar K, Ng PK (2018). Comprehensive molecular characterization of the Hippo Signaling Pathway in Cancer. Cell Rep.

[30] Asan U, Agency BCC, Brigham, Women’s H, Broad I, Cancer Genome Atlas Research N, Analysis Working Group (2017). Integrated genomic characterization of oesophageal carcinoma. Nature.

[31] Berger AC, Korkut A, Kanchi RS, Hegde AM, Lenoir W, Liu W (2018). A Comprehensive Pan-cancer Molecular Study of gynecologic and breast cancers. Cancer Cell.

[32] Lee BS, Park DI, Lee DH, Lee JE, Yeo MK, Park YH (2017). Hippo effector YAP directly regulates the expression of PD-L1 transcripts in EGFR-TKI-resistant lung adenocarcinoma. Biochem Biophys Res Commun.

[33] Xie M, Zhang L, He CS, Hou JH, Lin SX, Hu ZH (2012). Prognostic significance of TAZ expression in resected non-small cell lung cancer. J Thorac Oncology: Official Publication Int Association Study Lung Cancer.

[34] Malik SA, Khan MS, Dar M, Hussain MU, Mudassar S (2017). TAZ is an independent prognostic factor in non-small cell lung carcinoma: elucidation at protein level. Cancer Biomark.

[35] Totaro A, Zhuang Q, Panciera T, Battilana G, Azzolin L, Brumana G (2019). Cell phenotypic plasticity requires autophagic flux driven by YAP/TAZ mechanotransduction. Proc Natl Acad Sci USA.

[36] Pavel M, Renna M, Park SJ, Menzies FM, Ricketts T, Füllgrabe J (2018). Contact inhibition controls cell survival and proliferation via YAP/TAZ-autophagy axis. Nat Commun.

[37] Barthet VJA, Brucoli M, Ladds M, Nössing C, Kiourtis C, Baudot AD et al. Autophagy suppresses the formation of hepatocyte-derived cancer-initiating ductular progenitor cells in the liver. Sci Adv. 2021;7(23). 10.1126/sciadv.abf9141 PMC8177709 34088666

[38] Marsh T, Debnath J (2020). Autophagy suppresses breast cancer metastasis by degrading NBR1. Autophagy.

[39] Cai J, Li R, Xu X, Zhang L, Lian R, Fang L (2018). CK1alpha suppresses lung tumour growth by stabilizing PTEN and inducing autophagy. Nat Cell Biol.

[40] Humpton TJ, Alagesan B, DeNicola GM, Lu D, Yordanov GN, Leonhardt CS (2019). Oncogenic KRAS induces NIX-Mediated Mitophagy to promote pancreatic Cancer. Cancer Discov.

[41] White E (2015). The role for autophagy in cancer. J Clin Invest.

[42] Russell RC, Guan KL (2022). The multifaceted role of autophagy in cancer. EMBO J.

[43] Nazio F, Bordi M, Cianfanelli V, Locatelli F, Cecconi F (2019). Autophagy and cancer stem cells: molecular mechanisms and therapeutic applications. Cell Death Differ.

[44] Miao CC, Hwang W, Chu LY, Yang LH, Ha CT, Chen PY (2022). LC3A-mediated autophagy regulates lung cancer cell plasticity. Autophagy.

[45] Chen X, Mao R, Su W, Yang X, Geng Q, Guo C (2020). Circular RNA circHIPK3 modulates autophagy via MIR124-3p-STAT3-PRKAA/AMPKα signaling in STK11 mutant lung cancer. Autophagy.

