# Supplementary material for: RGS20 promotes non-small cell lung carcinoma proliferation via autophagy activation and inhibition of the PKA-Hippo signaling pathway
Source: Cancer Cell Int. 2024 Mar 2;24:93. doi: 10.1186/s12935-024-03282-9 (PMC10909273; doi:10.1186/s12935-024-03282-9)
Supplement: Supplementary file 1 — Supplementary Material 1 [file 12935_2024_3282_MOESM1_ESM.docx]

Supplement Table 1: The human *rgs20* shRNA sequences used in lentiviruses and the primer sequences of *rgs20 used* in q-PCR

| Name | Sequences |
| --- | --- |
| shRNA-1 | CGGAGAAATCTATTGAAGCAT |
| shRNA-2 | CCATCCCAACACATATTCGAT |
| shRNA-3  *rgs20*  *gapdh* | GCTCAGTCATTTGACAAATTA  F-CAGATGGGATCAGAGCGGATG  R-GGTTTCTAACAGTGAGACACGAG  F-GGTCACCAGGGCTGCTTTTA  R-GGATCTCGCTCCTGGAAGATG |
