# Supplementary material for: RGS20 promotes non-small cell lung carcinoma proliferation via autophagy activation and inhibition of the PKA-Hippo signaling pathway
Source: Cancer Cell Int. 2024 Mar 2;24:93. doi: 10.1186/s12935-024-03282-9 (PMC10909273; doi:10.1186/s12935-024-03282-9)
Supplement: Supplementary file 2 — Supplementary Material 2 [file 12935_2024_3282_MOESM2_ESM.docx]

Supplement Table 2: The antibodies information used in immunoblotting, IF and IHC.

| Name Catalog Number | Manufacturer Dilution |
| --- | --- |
| RGS20 ab239030 | Abcom 1:1000 |
| GAPDH 60004-1-lg | Proteintech 1:5000 |
| LC3A 12319 | ABclonal 1:1000 |
| P62 A19700 | ABclonal 1:1000 |
| Beclin-1 #3495 | CST 1:1000 |
| YAP #14047  p-YAP (Ser127) #13008  p-PKA (Thr197) #5661  PKA #5842 | CST 1:1000  CST 1:1000  CST 1:1000  CST 1:1000 |
| HRP-labeled Goat Anti-Mouse IgG | Epizyme 1:5000 |
| HRP-labeled Goat Anti- Rabbit IgG  YAP (IF) #14047 | Epizyme 1:5000  CST 1:100 |
| RGS20(IHC) ab239030 | Abcom 1:200 |
| Ki67(IHC) 30-9  YAP(IHC) #14047  p-YAP(IHC) #13008  PKA(IHC) #5842  p-PKA(IHC) #5661 | Ventana 1:200  CST 1:200  CST 1:200  CST 1:200  CST 1:200 |

IF: Immunofluorescence IHC: immunohistochemistry
