# Supplementary material for: RGS20 promotes non-small cell lung carcinoma proliferation via autophagy activation and inhibition of the PKA-Hippo signaling pathway
Source: Cancer Cell Int. 2024 Mar 2;24:93. doi: 10.1186/s12935-024-03282-9 (PMC10909273; doi:10.1186/s12935-024-03282-9)
Supplement: Supplementary file 3 — Supplementary Material 3 [file 12935_2024_3282_MOESM3_ESM.docx]

**Supplement Figure 1 A** Representative Western blot image to show RGS20 protein level in three NSCLC cell lines. GAPDH was used as a loading control. The quantification analysis was shown at the right. All results were expressed as the mean ± SD (n = 3). *P < 0.05. **B** The mRNA expression of rgs20 in stable transfected H1299 and Anip973 cell lines was examined by q-PCR. Results were presented as the mean ± SD (n = 3). ***p<0.001. Representative Western blot image of RGS20 to show the protein level after overexpression **(C)** or knockdown (shRNA-1 and shRNA-3) **(D) in** H1299 and Anip973 cell lines. Protein levels were normalized to GAPDH. Results were presented as the mean ± SD (n = 3). ***p<0.001.

**
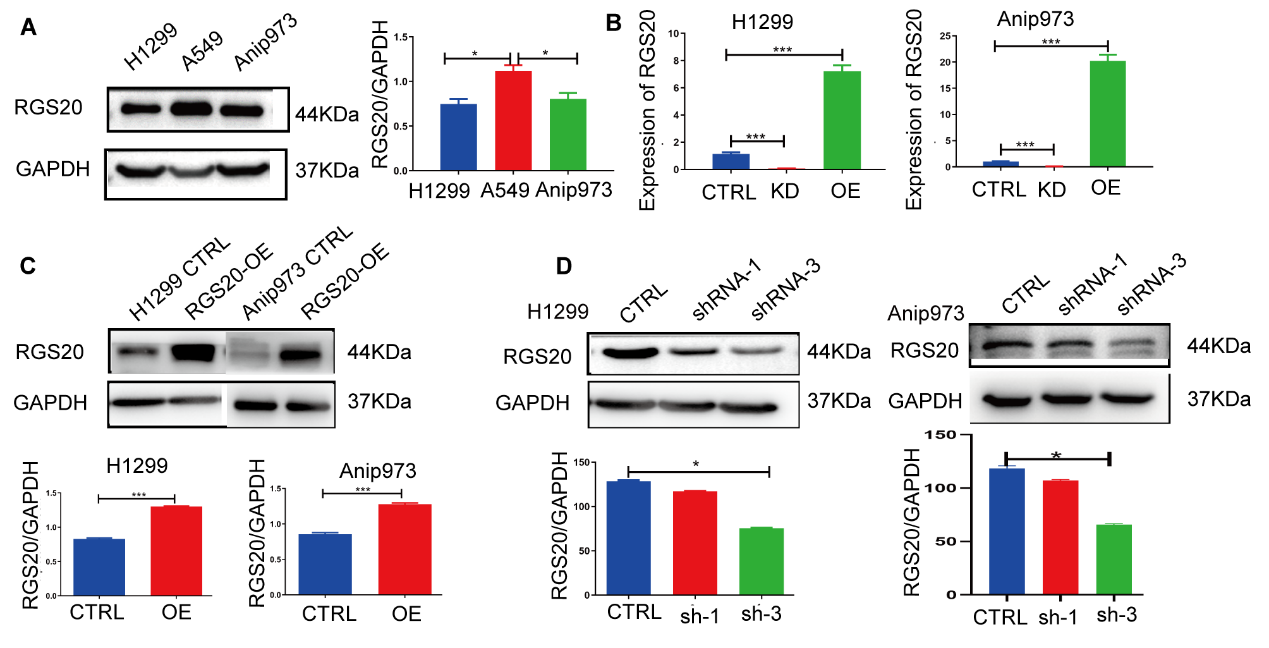
**
