# Supplementary material for: RGS20 promotes non-small cell lung carcinoma proliferation via autophagy activation and inhibition of the PKA-Hippo signaling pathway
Source: Cancer Cell Int. 2024 Mar 2;24:93. doi: 10.1186/s12935-024-03282-9 (PMC10909273; doi:10.1186/s12935-024-03282-9)

**Supplement Figure 2 A** The heat map analysis showed the gene expression differences between RGS20-OE group and the control group in H1299 cells. The top 10 up- and down-regulated mRNAs were listed by transcriptome sequencing. **B** GO enrichment result to show the biological processes of which the differentially expressed genes were enriched in.


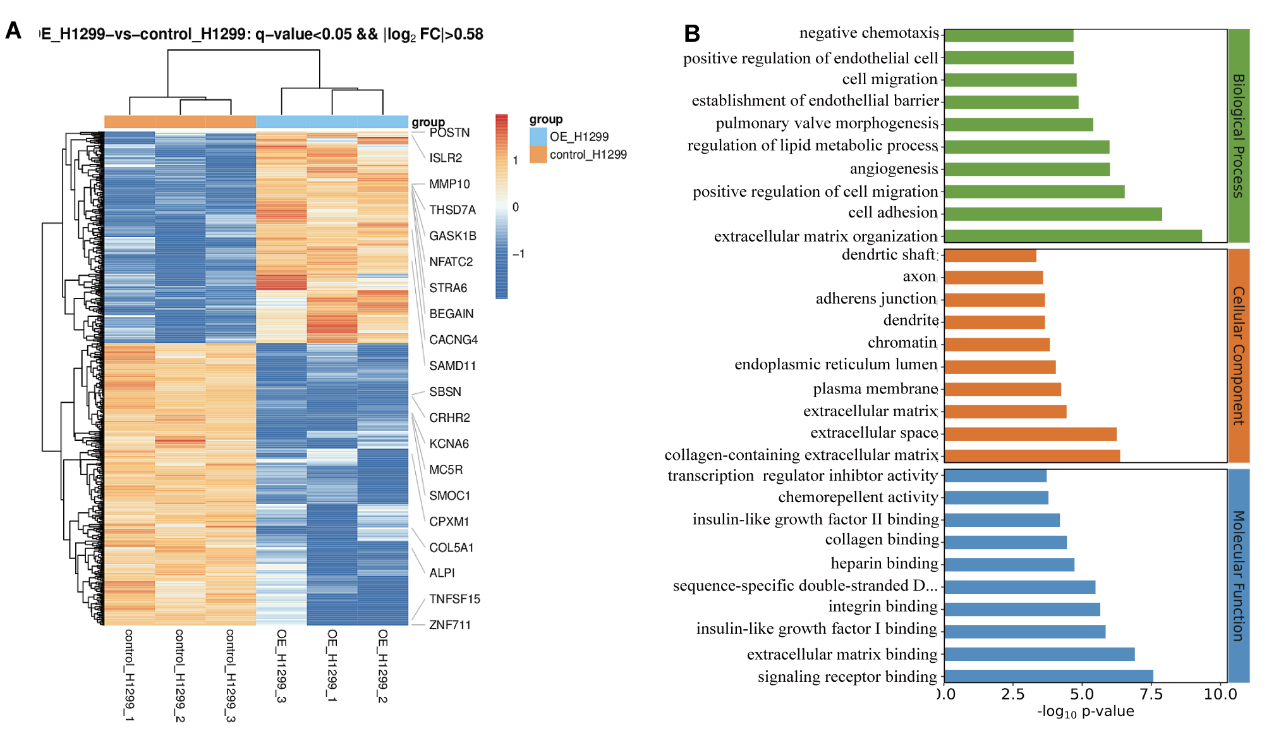

Supplement: Supplementary file 4 — Supplementary Material 4 [file 12935_2024_3282_MOESM4_ESM.docx]
