# Supplementary figures and images for: RGS20 promotes non-small cell lung carcinoma proliferation via autophagy activation and inhibition of the PKA-Hippo signaling pathway
Source: Cancer Cell Int. 2024 Mar 2;24:93. doi: 10.1186/s12935-024-03282-9 (PMC10909273; doi:10.1186/s12935-024-03282-9)

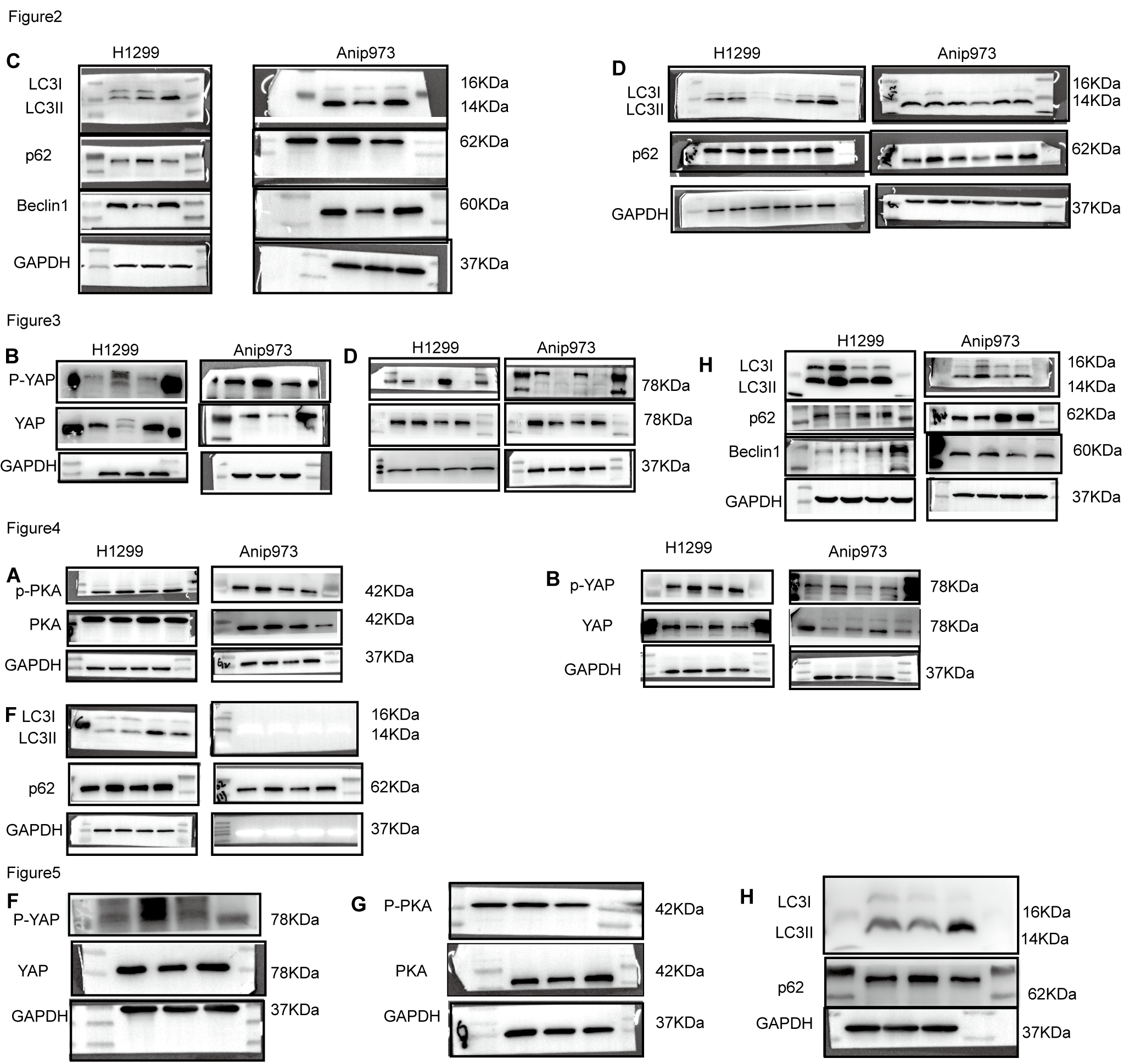

Supplement: Supplementary file 5 — Supplementary Material 5 [file 12935_2024_3282_MOESM5_ESM.png]
